# CKATool: A clinical kinematic analysis toolbox for upper limb rehabilitation

**DOI:** 10.1016/j.csbj.2025.08.018

**Published:** 2025-08-27

**Authors:** Ummi K. Latif, Georgi V. Georgiev

**Affiliations:** Center for Ubiquitous Computing, University of Oulu, Oulu, 90570, Northern Ostrobothnia, Finland

**Keywords:** Kinematic analysis, Upper-limb assessment, Software, Toolbox

## Abstract

This study introduces CKATool, the Clinical Kinematic Analysis Toolbox, a Python-based tool for analyzing upper limb movement using 3D motion tracking data. CKATool implements a comprehensive set of commonly used kinematic metrics in rehabilitation and movement science, organized into six domains: speed, smoothness, efficiency, accuracy, control strategy, and joint range of motion. Each metric is implemented as a separate function, allowing users to easily apply, modify, or extend the analysis based on specific research or clinical needs. CKATool integrates the Rerun platform for fast, interactive 3D visualization of joint trajectories and metric outcomes. The toolbox was evaluated using three publicly available datasets: Arm-CODA, 3D-ARM-Gaze, and IntelliRehabDS, spanning both structured and naturalistic upper limb tasks. CKATool successfully processed and visualized all datasets, demonstrating its compatibility with different types of experiments and rehabilitation setups. The source code, documentation, and data formatting scripts are freely available to support open and reproducible research.

## 1 Introduction

Quantitative assessment of upper limb motor function is essential for advancing our understanding of human movement and motor control. In movement science research and rehabilitation contexts, precise measurement of movement quality is crucial for evaluating motor recovery, quantifying impairments, and guiding therapy. Impairments caused by neurological and musculoskeletal conditions, such as stroke, cerebral palsy, or traumatic brain injury, can interfere with the smooth and coordinated execution of goal-directed movements. Kinematic analysis is a valuable method for detecting and monitoring these impairments, providing objective and high-resolution insights into movement patterns over time. Often, specific metrics are emphasized based on the clinical population. For example, smoothness and accuracy are frequently used to assess recovery in stroke patients [1], [2], range of motion (ROM) and endpoint trajectory are particularly relevant for individuals with cerebral palsy [3], [4]. Movement variability and inter-joint coordination measurement can be necessary for traumatic brain injury [5], [6].

Obtaining such clinically relevant metrics depends on the accuracy and reliability of the movement data captured by motion tracking technology. Optoelectronic motion capture systems are often considered the gold standard for precision, enabling detailed tracking of joint trajectories but requiring specialized facilities and setup [2]. Inertial measurement units (IMUs) offer a portable, lower-cost alternative suitable for home-based rehabilitation, though they may be sensitive to drift [7], [8]. Depth cameras provide markerless tracking and greater accessibility, but with reduced accuracy under certain conditions [9], [10]. Virtual reality (VR) based tracking systems, including head-mounted displays and hand controllers, allow simultaneous measurement and task engagement in immersive environments, offering unique opportunities for functional assessment. At the same time, their accuracy may be affected by occlusion, limited tracking volume, or controller placement [11], [12]. In some cases, some researchers develop their own custom motion tracking systems to suit their specific requirements [13], [14]. The choice of technology is often influenced by the clinical population, the intended metrics, and practical constraints.

Despite advances in both clinical application and motion capture technology, there remains a gap in integrating clinically validated kinematic metrics into accessible, standardized software. Several widely used platforms, such as OpenSim [15], Biomechanical ToolKit [16], and Kinematic Assessment Tool (KAT) [17], provide valuable capabilities for musculoskeletal modeling, biomechanical data handling, or planar motor assessments. However, these systems are not specifically designed to generate standardized, clinically interpretable kinematic metrics for upper limb rehabilitation. Other motion analysis tools require custom scripting [18] or are designed primarily for research contexts [19], [20], limiting adoption in routine clinical practice. This fragmentation results in inconsistent metric definitions, inefficient data processing, and reduced reproducibility across studies. [21].

In response to these challenges, we developed CKATool (Clinical Kinematic Analysis Toolbox), a modular and extensible software solution designed for computing and visualizing upper limb kinematic metrics from 3D motion tracking data. CKATool includes a comprehensive set of clinically validated metrics across six domains: speed, smoothness, efficiency, accuracy, control strategy, and joint ROM. This allows for flexible and condition-specific analysis. Designed to be compatible with multiple motion tracking systems, CKATool provides sample scripts as a guideline for input data formatting. Additionally, it features high-performance visualizations for joint trajectories and metric outcomes. By offering a standardized and adaptable framework, CKATool aims to bridge the gap between research-grade kinematic analysis and clinically meaningful assessments.

## 2 Toolbox description

CKATool is a Python-based system designed to compute standardized and literature-supported kinematic metrics from 3D upper limb movement data. Developed to support both rehabilitation research and movement analysis, CKATool offers modular and extensible functions that can be tailored to a wide range of experimental designs, sensor setups, and data pipelines. It is particularly well-suited for analyzing repeated reaching or task-based upper limb movements recorded using motion capture systems. The toolbox's architecture allows researchers and clinicians to select or adapt analysis components based on specific study goals or system configurations. To support this flexibility, the implementation relies on a structured and transparent design, as described below.

### 2.1 Implementation

CKATool was developed in Python and is designed to process 3D positional data collected from seven motion trackers placed on key upper limb anatomical landmarks: the wrist, elbow, and shoulder on both the left and right sides, as well as the trunk and neck. If neck position data is not provided, it can be algorithmically estimated based on the spatial relationship between the shoulders and the trunk.

Input data must be provided in CSV format, containing labeled time-series data for the relevant joints. A schema template is included in CKATool to ensure consistent formatting. Users can reformat data from publicly available datasets or motion capture systems to match this specification.

Once validated and segmented, CKATool processes the data using a set of independent metric functions, each designed to extract a specific kinematic property. These functions are based on well-established computational approaches from prior research, enabling users to generate interpretable, quantitative measures aligned with standard assessment practices.

The metrics are organized into six primary domains commonly used in upper limb kinematic evaluation: speed [12], [22], [23], [24], smoothness [22], [25], [26], [27], efficiency [27], [28], [29], [30], accuracy [24], [28], control strategy [27], [30], [31], and joint ROM [19], [20], [32], [33]. Each domain contains multiple metric functions, following the domain-based classification introduced by Ana et al. [34]. CKATool is designed to compute all available metrics in a single execution. However, because each metric is implemented as a separate function in the source code, researchers can selectively disable or include specific metrics by modifying the relevant code segments, and can extend the toolbox with additional, custom-defined metrics if required.

### 2.2 Visualization and interface

To enable intuitive exploration of motion data and analysis results, CKATool integrates the open-source Rerun visualization tool [35]. Rerun offers a high-performance, interactive interface that runs entirely on the local machine, ensuring responsiveness and data privacy. It supports synchronized 3D visualization of joint trajectories alongside relevant kinematic metrics, enabling users to examine both spatial and temporal aspects of movement within a unified environment.

The interface allows users to:

• Step through motion sequences frame by frame,
• Display joint trajectories alongside metric values such as velocity, smoothness, and range of motion, enabling detailed analysis of movement characteristics,
• Facilitates visual comparison of movement characteristics across repetitions and between limbs within a single recording.

This integration of visualization and metrics enhances the interpretation of joint-level behavior during each trial, enabling both qualitative and quantitative analysis. While CKATool does not currently include built-in export functions for metrics, all results are accessible via Python objects and can be logged manually or integrated into custom data processing pipelines. The modular design and use of Rerun provide a flexible foundation that can be easily extended to support additional visualization needs or workflow customizations, making the toolbox particularly well-suited for research and clinical prototyping contexts.

### 2.3 System requirements

CKATool is compatible with Python 3.11 or later and can be run on Windows and Linux systems. It is built using widely adopted Python libraries for numerical computation, data processing, and visualization, including NumPy, Polars, scikit-learn, and Rerun.

To simplify installation and dependency management, the toolbox supports setup using the ‘uv’ Python package manager, a modern alternative to ‘pip’ that ensures fast, reproducible, and isolated environments. A detailed README is included in the repository, providing step-by-step setup instructions, environment configuration, and example commands for running the toolbox with sample data.

## 3 Kinematic metrics overview

CKATool provides a library of modular functions for computing key kinematic metrics from 3D upper limb motion data. These metrics are organized into six domains commonly referenced in the rehabilitation and motor control literature [34]. All metrics are implemented using established mathematical definitions to ensure comparability with previous research. This section outlines the available metrics and their computational formulas when applicable.

While the computational structure of many metrics is shared, their interpretation depends on the type of input data. CKATool supports both linear kinematics, derived from 3D positional data (e.g., hand or wrist trajectories), and angular kinematics, derived from joint angle time series (e.g., shoulder or elbow rotation). This dual capability allows users to analyze movement quality at segmental and joint levels within a unified analytical framework.

These kinematic metrics also hold clinical significance as indicators of motor function, recovery, or impairment in rehabilitation contexts. For example, ROM is a standard clinical measure of joint mobility, while movement speed and smoothness are associated with neuromuscular coordination and control [36], [37]. By providing high-resolution, quantitative assessments of these features, CKATool can complement established outcome measures such as the Fugl-Meyer Assessment [38] or the DASH (Disabilities of the Arm, Shoulder, and Hand) questionnaire [39], assisting clinicians and researchers in monitoring patient progress with greater precision.

### 3.1 Speed

Speed-related metrics offer a fundamental yet valuable assessment of movement performance. They quantify how fast a movement is performed and are commonly used to evaluate motor function, monitor rehabilitation progress, and compare performance across different task conditions. CKATool includes three key metrics to characterize the speed profile of upper limb movement.

#### 3.1.1 Movement time

Movement Time (MT) is defined as the total duration required to complete a single movement segment. In this toolbox, movement onset ($t_{0}$) and offset ($t_{1}$) are determined based on the segmented intervals provided in the input data. The metric is calculated as:

(1) $$\text{MT}=t_{1}-t_{0}$$

This measure indicates the time required for a complete movement iteration, commonly utilized to evaluate speed, control, and performance efficiency, especially in rehabilitation and motor assessment contexts.

#### 3.1.2 Mean velocity (MV)

Mean Velocity (MV) represents the average speed during a movement segment. It was calculated by taking the mean of all speed values within each segment's speed profile.

(2) $$\text{MV}=\frac{1}{N}\sum_{i=1}^{N}v_{i}$$

where $v_{i}$ is the instantaneous speed at time point *i*, and *N* is the total number of time points in the segment.

#### 3.1.3 Peak velocity

Peak velocity (PV) represents the maximum speed reached during a movement segment. It was calculated by identifying the highest value in the speed profile for that segment.

(3) $$\text{PV}=\max\left(v_{1},v_{2},\ldots ,v_{N}\right)$$

where $v_{i}$ is the instantaneous speed at time point *i*, and *N* is the total number of time points in the segment.

### 3.2 Smoothness

Smoothness is a fundamental characteristic of well-coordinated motion, reflecting the fluidity and efficiency of motor control. It is often used to evaluate motor learning and recovery, especially in clinical and rehabilitation contexts. Over the years, several metrics have been widely adopted to assess movement smoothness. The following subsections introduce and explain these widely utilized smoothness measures.

#### 3.2.1 Ratio mean and peak velocity

The *ratio of mean to peak velocity* is a smoothness metric that shows how evenly the speed is distributed during a movement. It compares the average speed to the highest (peak) speed in each movement segment. A higher ratio means the speed stayed more consistent, which is usually a sign of smoother movement. A lower ratio suggests the movement had a quick burst of speed followed by slower parts, which may indicate stops, corrections, or less control.

This metric is calculated for each movement segment, allowing for a segment-by-segment assessment of smoothness throughout a task or trial. Since it normalizes mean speed by the peak, it provides a unitless value that enables comparison across segments of different durations and magnitudes.

(4) $$\text{Velocity Ratio}=\frac{MV}{PV}$$

#### 3.2.2 Number of peak velocity

The Number of Peak Velocity (NPV) refers to the count of local maxima in the speed profile over time. A higher number of peaks typically indicates more frequent interruptions or submovements within the motion, reflecting reduced smoothness and lower coordination. A peak is identified by finding points in the speed profile where the speed increases and then decreases, forming a local maximum.

A velocity peak at time $t_{i}$ was detected if the following conditions were satisfied:

1. $v(t_{i})$ is a local maximum:
  $$v(t_{i-1})<v(t_{i})>v(t_{i+1})$$
2. The amplitude difference between the peak and the preceding local minimum $v(t_{\text{min}})$ exceeds a velocity threshold δ=20 mm/s:
  $$v(t_{i})-v(t_{\text{min}})>\delta$$
3. The time interval from the previous detected peak $t_{i-1}^{\text{peak}}$ is at least τ=150 ms:
  $$t_{i}-t_{i-1}^{\text{peak}}\geq \tau$$

The choice of these parameters and peak detection criteria was guided by previous studies [40], [41] and visual inspection of speed profiles derived from the kinematic data. However, the threshold values can be adjusted based on the characteristics of the speed profiles observed in the dataset.

#### 3.2.3 Zero-crossings in acceleration

Zero-crossings in acceleration quantify movement smoothness by counting how often the acceleration signal changes direction (i.e., crosses zero). As defined by Lum et al. [42], this metric was originally applied to the tangential acceleration profile of the hand path. A higher number of zero-crossings is associated with reduced smoothness, indicating more corrective submovements.

This method can be applied to both linear and angular movement profiles. For linear motion, it is based on the second derivative of position $\overset{¨}{x}(t)$, and for angular motion, on the second derivative of joint angle $\overset{¨}{\theta }(t)$. The metric is computed as:

(5) $${\text{ZC}}_{\text{acc}}=\sum_{i=1}^{N-1}1\left[{\overset{¨}{s}}_{i}\cdot {\overset{¨}{s}}_{i+1}<0\right]$$

where $\overset{¨}{s}$ is the acceleration signal (linear or angular), and 1[⋅] is an indicator function that returns 1 when the sign of acceleration changes between consecutive time steps.

#### 3.2.4 Jerk

Jerk-based metrics are commonly used to quantify movement smoothness by measuring the rate of change of acceleration over time. In this work, we implement the *Log Dimensionless Jerk (LDJ)* to evaluate smoothness from 3D position data. LDJ is calculated by computing the third derivative of position (jerk), integrating the squared jerk magnitude over the movement duration, normalizing it by movement time and path length, and then applying a negative natural logarithm to produce a scale-invariant, unitless measure. We chose LDJ instead of the traditional *Normalized Jerk* because it is more sensitive to detecting subtle changes in movement quality and less affected by signal noise or pauses during motion [17], [43]. The LDJ is computed using the following equation:

(6) $$\text{LDJ}=-\ln\left(\frac{1}{PL^{2}MT^{5}}\int_{0}^{T}j{\left(t\right)}^{2}\;dt\right)$$

where j(t) is the magnitude of the third derivative of position (jerk), *MT* is the total movement time or duration, and *PL* is the path length.

#### 3.2.5 Spectral arc-length

Spectral arc-length (SPARC) is a measure of movement smoothness based on the frequency content of the speed profile v(t). It evaluates how much the shape of the frequency spectrum changes. Smoother movements tend to have simpler, more concentrated frequency content, resulting in a smaller (more negative) arc length value. To compute SPARC, the speed signal is transformed into the frequency domain. The magnitude of this spectrum is normalized and used to calculate the arc length across a frequency range up to a cut-off $\omega_{c}$. The metric is defined as:

(7) $$\text{SPARC}≜-\int_{0}^{\omega_{c}}\sqrt{{\left(\frac{1}{\omega_{c}}\right)}^{2}+{\left(\frac{d\overset{ˆ}{V}(\omega )}{d\omega }\right)}^{2}}\;d\omega$$

(8) $$\overset{ˆ}{V}(\omega )≜\frac{V(\omega )}{V(0)}$$

where $\overset{ˆ}{V}(\omega )=\frac{V(\omega )}{V(0)}$ is the normalized Fourier magnitude spectrum of the speed signal, and $\omega_{c}$ (typically 40*π* rad/s or 20 Hz) defines the frequency band relevant to human movements. In CKATool analysis, the SPARC calculation was implemented using the publicly available code from https://github.com/siva82kb/SPARC, which is based on the method described by Balasubramanian et al. [44]. A larger (less negative) SPARC value indicates smoother and more coordinated motion, while a smaller (more negative) value reflects jerkier or less smooth movement.

### 3.3 Efficiency

The efficiency domain evaluates how economically a movement is executed, focusing on whether the path taken to reach a target is direct or contains unnecessary deviations. Efficient movement is often associated with better motor planning and execution, while inefficient trajectories may reflect compensatory strategies or motor impairments. A commonly used metric in this domain is the hand path ratio, which compares the actual trajectory to the ideal path.

#### 3.3.1 Hand path ratio

Hand Path Ratio (HPR) is a commonly used metric to assess movement efficiency by comparing the actual path taken by the hand to reach a target with the shortest possible (straight-line) path. It quantifies how directly a participant moved toward the target. The formula is:

(9) $$\text{HPR}=\frac{\text{Actual path length}}{\text{Straight-line distance}}.$$

An optimal and completely efficient motion would produce a HPR value of 1.0, signifying that the hand proceeded straight to the target without any deviations. Values exceeding 1 suggest a less efficient trajectory, often caused by adjustments, hesitations, or non-ideal paths. This measurement is especially important in rehabilitation settings, where it is crucial to reduce unnecessary movements.

### 3.4 Accuracy

The accuracy domain focuses on how precisely a movement reaches its intended goal. These metrics evaluate spatial closeness to the target and are essential for assessing task performance, motor control, and movement quality in both healthy and clinical populations. One of the most widely used accuracy metrics is target error, which quantifies how close the end-effector comes to the desired endpoint.

#### 3.4.1 Target error

Target error assesses the spatial precision of movement by determining the Euclidean distance from the end-effector's (such as the hand's) final position to the desired target location. It reflects the accuracy with which the participant attained the target. The error is computed as:

(10) $$\text{Target error}=\|p_{\text{final}}-p_{\text{target}}\|,$$

where $p_{\text{final}}$ is the position of the hand at the end of the movement, and $p_{\text{target}}$ is the designated target location. Reduced error values indicate improved accuracy, whereas greater errors highlight challenges in reaching or accurately stopping at the target. This measure is crucial for assessing motor control and progress in rehabilitation.

### 3.5 Control strategy

The control strategy domain provides insights into how movement is planned and executed over time. These measures go beyond outcome-based metrics like speed or accuracy by capturing the temporal and dynamic features of motor control. One commonly used metric in this domain is time to peak velocity, which reflects how quickly peak movement speed is reached during a motion.

#### 3.5.1 Time to peak velocity

Time to Peak Velocity (TPV) represents how early the peak speed occurs during a movement. A lower TPV indicates a quick burst of speed followed by a longer correction phase, which is common in precise, goal-directed tasks [45]. To allow comparison across movements of different durations, TPV is expressed as a percentage of the total movement time. It is calculated using the following formula:

(11) $$\text{TPV}\;[\text{\%}]=\left(\frac{t_{\text{peak}}-t_{\text{start}}}{t_{\text{end}}-t_{\text{start}}}\right)\times 100$$

where $t_{\text{peak}}$ is the time of peak speed, $t_{\text{start}}$ is the movement start time, and $t_{\text{end}}$ is the movement end time.

### 3.6 Joint range of motion

Joint range of motion (ROM) quantifies the angular displacement of a joint during movement and serves as a key measure of physical ability and motor function. In our system, we compute ROM over time to observe the extent of movement for each joint during the entire task duration.

To calculate the ROM at the elbow, we utilize the positions of the wrist, elbow, and shoulder joints. The angle at the elbow is determined by measuring the angle between the upper arm vector (extending from the shoulder to the elbow) and the forearm vector (extending from the elbow to the wrist).

Shoulder ROM is determined by the relative positions of the shoulder, elbow, and a reference point on the trunk. To maintain consistency among participants and datasets, a virtual trunk point is created by horizontally aligning it with the shoulder, following the direction from the neck to the shoulder. The shoulder angle is then calculated as the angle between the upper-arm vector (from the shoulder to the elbow) and the trunk vector (from the shoulder to the virtual trunk point). This procedure is similar to how physical therapists assess shoulder ROM in clinical practice. By using anatomical landmarks such as the sternum (chest), clavicle (collarbone), and scapula (shoulder blade), therapists align a goniometer to measure the angle between the trunk and the arm [46]. Our method digitally replicates this process by utilizing tracked joint positions in 3D space.

Although trunk-based references help standardize shoulder angle computation, posture-related factors, such as pelvic tilt or trunk compensation, may still introduce minor variability in ROM measurements, as noted in prior clinical studies [47].

By standardizing these calculations for both elbow and shoulder joints, CKATool ensures consistent ROM measurements across datasets, supporting reliable assessment of joint mobility and movement capacity in rehabilitation contexts.

## 4 Usability assessment

This section presents the usability evaluation and functional demonstration of CKATool. To ensure clinical and research applicability, CKATool was tested on multiple upper limb motion datasets with distinct task designs and recording contexts. The evaluation aimed to assess input compatibility, metric computation, visualization functionality, and practical responsiveness under real-world conditions.

### 4.1 Evaluation strategy

To validate the functionality and generalizability of CKATool, we evaluated it using three publicly available datasets containing 3D upper limb motion data from both healthy individuals and patients: the Arm-CODA dataset [48], the 3D ARM Gaze dataset [49], and the IntelliRehabDS dataset [50]. These datasets were selected for their diverse characteristics, offering a comprehensive testbed to assess CKATool's performance across various task types, sensor modalities, and user populations.

As this study involves the secondary use of publicly available datasets for tool validation, all participant recruitment procedures, inclusion/exclusion criteria, and ethical approvals were handled by the original dataset providers. We refer readers to the respective publications for full methodological details. Key participant characteristics are summarized below to contextualize the evaluation.

The Arm-CODA dataset consists of 240 multivariate time series from 16 healthy participants (aged 23–65; 11 males, 5 females) performing 15 predefined upper limb movements, such as arm raising and combing hair, recorded using the Cartesian Optoelectronic Dynamic Anthropometer (CODA) motion analysis system (Charnwood Dynamics Ltd.) with 34 reflective markers. All participants had no known medical impairments and provided informed consent. The dataset includes detailed metadata such as participant demographics and precise annotations of movement onset and offset for at least two repetitions per task. This structured setup makes Arm-CODA ideal for benchmarking segmentation, classification, and analysis of constrained and repetitive movement patterns.

The 3D-ARM-Gaze dataset complements Arm-CODA by capturing naturalistic reaching behaviors in an immersive virtual environment. It contains over 2.5 million samples from 20 participants (aged 19–44; 6 males and 14 females) completing more than 14,000 pick and place tasks while seated in a standardized posture. All participants were able-bodied, right-handed, with normal or corrected-to-normal vision, and had no known mental or motor disorders. Data were recorded using an HTC Vive headset with trackers (HTC Corporation), and capturing both target and end-effector coordinates. This makes the dataset particularly suitable for evaluating accuracy and efficiency metrics. With over 200 repetitions per file and more than 100,000 rows in some sequences, it also provides a robust opportunity to assess CKATool's performance on large-scale datasets.

To examine CKATool's applicability in rehabilitation contexts, we also incorporated the IntelliRehabDS dataset, which includes nine rehabilitation gestures performed by 29 participants, including 15 patients and 14 healthy controls, captured using a Kinect motion-sensing input device (Microsoft Corporation). The average age of the patient group was 43 years, while the healthy control group had an average age of approximately 26 years. The patient group presented a diverse range of conditions, including spinal cord injuries, strokes, brain injuries, neurological conditions, limb injuries, and amputations (with prosthetic leg). Each sample includes 3D coordinates of 25 body joints along with annotations for gesture type, body posture (sitting or standing), and correctness labels. This dataset supports the analysis of movement quality and performance deviations in clinical populations.

Together, these three datasets offer complementary motion scenarios, ranging from structured laboratory tasks to natural interactions in virtual environments and practical rehabilitation exercises. This allows for a robust and comprehensive validation of CKATool across multiple domains of upper limb motion analysis.

### 4.2 Dataset compatibility and preprocessing

All three datasets, captured using motion tracking systems, provided raw 3D positional data for key upper limb joints such as the shoulder, elbow, and wrist. Prior to analysis, the data were reformatted to meet the input requirements of CKATool. This preprocessing step included the following:

• Standardizing column names for each joint axis (e.g., WRx, WRy, WRz),
• Converting timestamps to UNIX time for temporal consistency,
• Assigning iteration labels to differentiate submovements or repeated movement segments.

In the original datasets, iteration or repetition information was either provided in a separate annotation file or encoded in a format that was not directly compatible with the toolbox. To address this, we aligned the iteration metadata with the main motion data using timestamps and the structure of the experimental tasks. This process ensured that the resulting data format matched CKATool's expectations without modifying the original motion recordings.

For shoulder ROM calculations, trunk orientation was estimated using available trunk reference points from each dataset. In the Arm-CODA dataset, markers were placed on the apophysis of the T7 and L3 vertebrae to define the trunk segment. The 3D-ARM-Gaze dataset included a marker explicitly attached to the trunk. In the IntelliRehabDS dataset, we used the SpineMid joint provided by the Kinect's skeleton tracking system to approximate trunk orientation. These references allowed for consistent computation of shoulder angles relative to the trunk across datasets, without relying on lower body markers such as those on the hips or pelvis.

A dataset converter script has been developed and shared along with CKATool to support reproducibility and future applications. This script includes sample usage and can be adapted for other datasets with a similar structure.

While our evaluation used datasets collected in controlled environments with high-quality optical, depth, and VR-based tracking systems, CKATool's modular architecture also supports adaptation to more variable or noisy data sources such as IMUs, low-cost depth sensors, or smartphone-based motion capture. These cases might benefit from additional preprocessing techniques, such as filtering, denoising, or sensor fusion, applied before the metric computation process [51], [52], [53]. The preprocessing methods and parameters should be selected based on the characteristics and quality of the user's data to ensure optimal performance.

### 4.3 Metric extraction and visualization

Once reformatted, each dataset was processed using CKATool's full analytical pipeline. All six metric domains: speed, smoothness, efficiency, accuracy, control strategy, and joint ROM, were successfully computed.

To support intuitive interpretation of results, visualizations were generated using the open-source Rerun visualization tool [35]. The visualizations highlight key movement characteristics and facilitate comparison across limbs, repetitions, and participant groups.

**Fig. 1:**
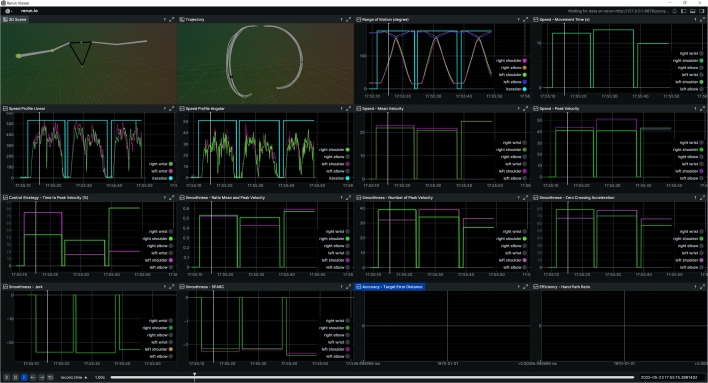
CKATool's visualization from the Arm-CODA dataset: scapular plane elevation performed in three repetitions by both arms.

Fig. 1 presents an example from the Arm-CODA dataset, showing a participant performing scapular plane elevation in a standing position. In this session, the participant completes three repetitions with both the right and left arms. The Range of Motion tab in visualization clearly shows that the shoulder joints exhibit the highest angular change, which aligns with the nature of the movement. Additionally, a difference in smoothness is observed between the two arms, with the right arm showing slightly smoother motion. This may be attributed to hand dominance, as the participant is right-handed.

**Fig. 2:**
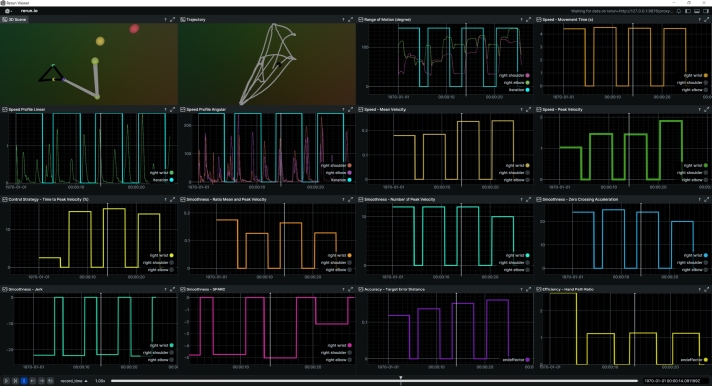
CKATool's visualization from the 3D ARM Gaze dataset, showing four repetitions of a reaching task with available target and end-effector data for assessing accuracy and efficiency.

Fig. 2 shows the visualization from the 3D-ARM-Gaze dataset. In this task, participants perform two reaching actions per repetition, returning to a neutral starting position after each pair. For clarity, the visualization includes only four repetitions, although the system is capable of processing more than 200 repetitions from a single file. This dataset uniquely provides both target and end-effector data, enabling computation and visualization of efficiency and accuracy metrics. In this example, we focus on wrist motion, and data from the elbow and shoulder joints were intentionally deactivated (indicated in gray) to reduce visual complexity.

**Fig. 3:**
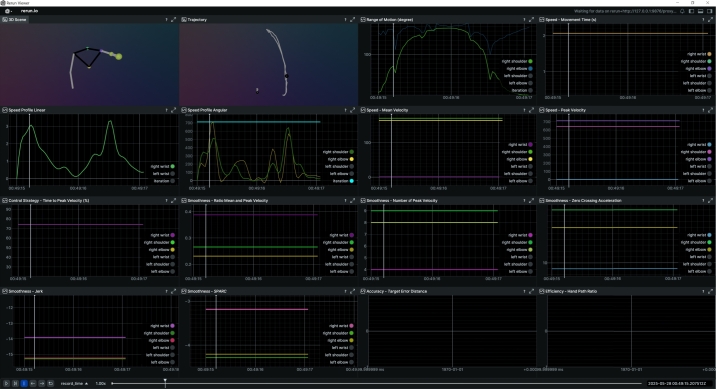
CKATool's visualization from IntelliRehabDS: healthy participant performing one repetition of shoulder flexion.

**Fig. 4:**
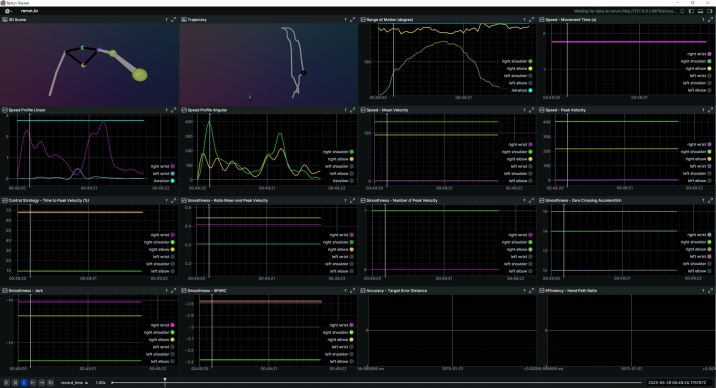
CKATool's visualization from IntelliRehabDS: patient participant with upper and lower limb weakness performing one repetition of shoulder flexion.

Fig. 3, Fig. 4 illustrate results from the IntelliRehabDS dataset, comparing a healthy participant and a patient with upper and lower limb weakness, respectively. Both are performing a shoulder flexion movement consisting of one full repetition (from rest to maximal flexion and back). The healthy participant exhibits a smoother trajectory, as evidenced by the movement path and smoothness metrics. The Range of Motion data also shows a greater flexion angle in the healthy individual. As expected, the patient's movement appears more restricted and less smooth. Since this dataset does not include target or end-effector data, accuracy and efficiency metrics could not be visualized.

Overall, these visualizations demonstrate the CKATool's ability to extract meaningful metrics and present them in an interpretable format across varied movement contexts and populations.

### 4.4 Performance profiling

To evaluate CKATool's computational performance, we measured both metric computation (Python processing) and interactive visualization (Rerun) using the three datasets employed for tool evaluation: 3D-ARM-Gaze, Arm-CODA, and IntelliRehabDS. For each dataset, five samples were randomly selected, and the average processing time and peak memory usage were recorded on a laptop equipped with an AMD Ryzen 9 5900HS processor, AMD Integrated Radeon Graphics, and 16 GB RAM. The results are summarized in Table 1.

**Table 1:** Summary of CKATool's runtime and memory usage across different datasets.

Dataset	File Size	Repetitions	Memory Usage (Python)	Memory Usage (Rerun)	Processing Time (s)
3D-ARM-Gaze	9700 KB	50	12.58 MB	653 MB	53.26
Arm-CODA	1655 KB	3	2.34 MB	379 MB	13.19
IntelliRehabDS	27 KB	1	0.373 MB	323 MB	2.33

During metric computation, peak memory usage remained low, ranging from 0.37 MB to 12.58 MB, with processing times between 2.33 s and 53.26 s depending on dataset size and number of repetitions.

For visualization, peak memory usage was higher (323 MB–653 MB), reflecting the cost of rendering and caching interactive 3D trajectories. This is expected behavior for real-time visualization tools. However, Rerun includes built-in settings for limiting RAM usage to suit different hardware configurations [54].

These results indicate that CKATool's metric computation is lightweight and scalable, and its visualization component can handle large datasets without excessive memory usage.

### 4.5 Limitation and future work

CKATool demonstrated strong performance and adaptability across diverse datasets. Key observations from the evaluation include:

• Seamless integration with motion capture data after minimal reformatting.
• High compatibility with various sensor formats, particularly for standard joint coordinate datasets.
• Computed metrics showed clear differences between structured and natural movements, confirming the validity of the analytical framework.
• Performance profiling showed fast processing and low memory usage for computation, with visualization demands manageable on typical hardware.

Despite these strengths, several limitations were identified:

• Manual mapping may still be necessary when dataset column names do not match the toolbox's expected naming format. However, we provide a sample data conversion script to help users reformat their datasets for CKATool compatibility.
• CKATool is currently designed for discrete movement analysis. It does not include functionality for automatic segmentation of continuous movement sequences. Therefore, input datasets must be pre-segmented into iterations before processing.
• There is no built-in export function for computed metrics. Although results are available through interactive visualization, exporting to structured formats (e.g., CSV or JSON) requires additional scripting.
• The current visualization layout was developed to reflect the computed metrics, but is not yet aligned with clinical assessment workflows. It does not incorporate domain-specific visual design guidelines commonly used by therapists or clinicians, nor does it currently support the inclusion of non-motor clinical information such as sensory or proprioceptive impairments, which are important for interpreting upper limb function in daily activities.

To improve usability and extend functionality, future development will focus on:

• Integrating automatic segmentation of continuous motion data into discrete movement iterations,
• Implementing export features for metrics in standardized file formats to facilitate downstream analysis and integration,
• Designing clinically relevant visualization layouts in collaboration with therapists or clinical professionals to better align with practical assessment needs, including the ability to incorporate contextual clinical information such as sensory or proprioceptive impairments.
• Developing a graphical user interface (GUI) to improve accessibility for non-technical users, such as clinicians and therapists, by enabling easier data input, metric selection, and interactive visualization.

While the introduction briefly notes the existence of other motion analysis tools such as OpenSIM, Biomechanical ToolKit, and KAT, the present study does not include a direct, systematic comparison of CKATool's features, performance, or outputs with these platforms. This omission limits our ability to position CKATool quantitatively within the broader landscape of motion analysis. Relatedly, to further strengthen its clinical utility and complement its technical capabilities, a future study will not only perform such cross-platform comparisons but also formally validate CKATool's computed kinematic metrics against established clinical outcome measures. These steps will help ensure the toolbox is competitive within the research landscape and aligned with real-world rehabilitation goals, supporting broader research and clinical practice adoption.

### 4.6 Security and accessibility

CKATool is designed for offline and local use, ensuring the privacy and security of sensitive motion data. All computations and visualizations are executed directly on the user's machine, with no data transmitted or stored externally. This design makes the toolbox suitable for deployment in secure clinical environments and compliant with institutional privacy policies.

Visualization is powered by the Rerun [35] open-source tool, which provides an interactive, real-time interface for exploring motion data. Since Rerun runs natively without requiring a web server or internet connection, it is well-suited for environments with strict network restrictions.

In addition, CKATool is cross-platform and does not require administrator privileges or specialized hardware. These characteristics make it accessible in low-resource settings, research laboratories, and rehabilitation clinics where access to technical infrastructure may be limited.

## 5 Conclusion

This paper introduced the CKATool (Clinical Kinematic Analysis Toolbox), a cross-platform tool designed to support the analysis and visualization of upper limb movement using 3D motion capture data. CKATool computes a comprehensive set of kinematic metrics across six domains: speed, smoothness, efficiency, accuracy, control strategy, and joint ROM. It provides researchers and clinicians with an accessible and extensible solution for evaluating movement quality in healthy individuals and clinical populations.

We applied CKATool's functionality and generalizability to three diverse, publicly available datasets: Arm-CODA, 3D-ARM-Gaze, and IntelliRehabDS. These datasets covered various use cases, from constrained laboratory tasks to naturalistic virtual reality interactions and rehabilitation exercises. After minimal reformatting, CKATool successfully processed all datasets and produced interpretable results aligned with expected motor behaviors.

Visualizations generated through the Rerun interface enabled intuitive exploration of joint trajectories and kinematic metric outputs, allowing for detailed comparisons across limbs and conditions. Because all processing occurs locally and offline, CKATool is well-suited for use in secure clinical environments where data privacy must be maintained.

Although the current version provides a strong foundation, some limitations remain. These include the need for pre-segmented input data and the absence of batch export functionality. Future updates will focus on incorporating automatic segmentation, more flexible input handling, and extended export features to support a broader range of workflows.

In summary, CKATool is a practical and versatile tool for rehabilitation research, human movement analysis, and clinical assessment. It has the potential to enhance both research practices and clinical decision-making.

## CRediT authorship contribution statement

**Ummi K. Latif:** Writing – review & editing, Writing – original draft, Visualization, Software. **Georgi V. Georgiev:** Writing – review & editing, Supervision, Conceptualization.

## Declaration of generative AI and AI-assisted technologies in the writing process

During the preparation of this work, the author(s) used ChatGPT to assist with drafting the manuscript and GitHub Copilot for tool development. After using this tool/service, the author(s) reviewed and edited the content as needed and take(s) full responsibility for the content of the publication.

## Declaration of Competing Interest

The authors declare that they have no known competing financial interests or personal relationships that could have appeared to influence the work reported in this paper.

## Data Availability

CKATool is freely available at https://github.com/khaira/ckatool. The repository includes comprehensive documentation, installation instructions, example input files, and data reformatting scripts to support reproducibility and facilitate adaptation to other datasets. CKATool was evaluated using three publicly available datasets: the Arm-CODA dataset [48], the 3D ARM Gaze dataset [49], and the IntelliRehabDS dataset [50]. These datasets can be downloaded from the sources provided in their respective publications. In addition, several raw samples and their corresponding reformatted versions are included in the toolbox repository. These are provided as references and for testing the applicability of the toolbox on different data types and structures.

## References

[1] Duret C., Courtial O., Grosmaire A.G. (2016). Kinematic measures for upper limb motor assessment during robot-mediated training in patients with severe sub-acute stroke. Restor Neurol Neurosci.

[2] Mesquita I.A., da Fonseca P.F.P., Pinheiro A.R.V., Correia M.F.P.V., da Silva C.I.C. (2019). Methodological considerations for kinematic analysis of upper limbs in healthy and poststroke adults part ii: a systematic review of motion capture systems and kinematic metrics. Top Stroke Rehabil.

[3] Jeffries L.M., LaForme Fiss A., Westcott McCoy S., Bartlett D., Avery L., Hanna S. (2019). Developmental trajectories and reference percentiles for range of motion, endurance, and muscle strength of children with cerebral palsy. Phys Ther.

[4] Klotz M.C., van Drongelen S., Rettig O., Wenger P., Gantz S., Dreher T. (2014). Motion analysis of the upper extremity in children with unilateral cerebral palsy—an assessment of six daily tasks. Res Dev Disabil.

[5] Ksenia P.I., Ustinova P.T. (2017). Decomposition of postural movements in individuals with mild tbi while reaching to intercept a moving virtual target. Physiother Theory Pract.

[6] Ustinova K.I., Langenderfer J.E., Balendra N. (2017). Enhanced arm swing alters interlimb coordination during overground walking in individuals with traumatic brain injury. Hum Mov Sci.

[7] Unger T., de Sousa Ribeiro R., Mokni M., Weikert T., Pohl J., Schwarz A. (2024). Upper limb movement quality measures: comparing imus and optical motion capture in stroke patients performing a drinking task. Front Digital Health.

[8] Hughes C.M.L., Tran B., Modan A., Zhang X. (2022). Accuracy and validity of a single inertial measurement unit-based system to determine upper limb kinematics for medically underserved populations. Front Bioeng Biotechnol.

[9] Scano A., Mira R.M., Cerveri P., Molinari Tosatti L., Sacco M. (2020). Analysis of upper-limb and trunk kinematic variability: accuracy and reliability of an rgb-d sensor. Multimodal Technol Interact.

[10] Lioulemes A., Theofanidis M., Makedon F. (2016). 2016 IEEE international conference on automation science and engineering.

[11] Latif U.K., Gong Z., Nanjappan V., Georgiev G.V. (2023). Proceedings of the design society, vol. 3.

[12] Everard G., Otmane-Tolba Y., Rosselli Z., Pellissier T., Ajana K., Dehem S. (2022). Concurrent validity of an immersive virtual reality version of the box and block test to assess manual dexterity among patients with stroke. J NeuroEng Rehabil.

[13] Soomro S.A., Nanjappan V., Georgiev G.V. (2023). Computer-aided design and applications, vol. 20.

[14] Barhoush Y., Georgiev G.V., Loudon B. (2020). Proceedings of the sixth international conference on design creativity (ICDC 2020).

[15] Seth A., Sherman M., Reinbolt J.A., Delp S.L. (2011). Opensim: a musculoskeletal modeling and simulation framework for in silico investigations and exchange. Proc IUTAM.

[16] Barre A., Armand S. (2014). Biomechanical toolkit: open-source framework to visualize and process biomechanical data. Comput Methods Programs Biomed.

[17] Culmer P.R., Levesley M.C., Mon-Williams M., Williams J.H. (2009). A new tool for assessing human movement: the kinematic assessment tool. J Neurosci Methods.

[18] Lapresa M., Zollo L., Cordella F. (2022). A user-friendly automatic toolbox for hand kinematic analysis, clinical assessment and postural synergies extraction. Front Bioeng Biotechnol.

[19] Arlati S., Keijsers N., Ferrigno G., Sacco M. (2020). 2020 IEEE international symposium on medical measurements and applications (MeMeA).

[20] Arlati S., Keijsers N., Paolini G., Ferrigno G., Sacco M. (2022). Kinematics of aimed movements in ecological immersive virtual reality: a comparative study with real world. Virtual Real.

[21] Di Matteo A., Lozzi D., Mignosi F., Polsinelli M., Placidi G. (2025). A dicom-based standard for quantitative physical rehabilitation. Comput Struct Biotechnol J.

[22] Evans J.O., Tsaneva-Atanasova K., Buckingham G. (2023). Using immersive virtual reality to remotely examine performance differences between dominant and non-dominant hands. Virtual Real.

[23] Barak Ventura R., Hughes K. Stewart, Nov O., Raghavan P., Ruiz Marín M., Porfiri M. (2022). Data-driven classification of human movements in virtual reality–based serious games: preclinical rehabilitation study in citizen science. JMIR Serious Games.

[24] Clark L.D., El Iskandarani M., Riggs S.L. (2023). Proceedings of the 2023 CHI conference on human factors in computing systems.

[25] Dong Y., Liu X., Tang M., Huo H., Chen D., Du X. (2023). Age-related differences in upper limb motor performance and intrinsic motivation during a virtual reality task. BMC Geriatr.

[26] Salisbury J.P., Aronson T.M., Simon T.J. (2020). Extended abstracts of the 2020 annual symposium on computer-human interaction in play.

[27] Levin M.F., Magdalon E.C., Michaelsen S.M., Quevedo A.A.F. (2015). Quality of grasping and the role of haptics in a 3-d immersive virtual reality environment in individuals with stroke. IEEE Trans Neural Syst Rehabil Eng.

[28] Knaut L.A., Subramanian S.K., McFadyen B.J., Bourbonnais D., Levin M.F. (2009). Kinematics of pointing movements made in a virtual versus a physical 3-dimensional environment in healthy and stroke subjects. Arch Phys Med Rehabil.

[29] Clark L., Riggs S. (2022). Extended abstracts of the 2022 CHI conference on human factors in computing systems.

[30] Magdalon E.C., Michaelsen S.M., Quevedo A.A., Levin M.F. (2011). Comparison of grasping movements made by healthy subjects in a 3-dimensional immersive virtual versus physical environment. Acta Psychol.

[31] Furmanek M.P., Schettino L.F., Yarossi M., Kirkman S., Adamovich S.V., Tunik E. (2019). Coordination of reach-to-grasp in physical and haptic-free virtual environments. J NeuroEng Rehabil.

[32] Jurado I.C., Rodríguez Vargas U., Penaloza C. (2020). 2020 IEEE international conference on systems, man, and cybernetics (SMC).

[33] Ginja G.A., Varoto R., Cliquet A. (2020). Proceedings of the 13th international joint conference on biomedical engineering systems and technologies (BIOSTEC 2020) - BIODEVICES.

[34] de los Reyes-Guzmán A., Dimbwadyo-Terrer I., Trincado-Alonso F., Monasterio-Huelin F., Torricelli D., Gil-Agudo A. (2014). Quantitative assessment based on kinematic measures of functional impairments during upper extremity movements: a review. Clin Biomech.

[35] Rerun Development Team (2025). Rerun: a visualization sdk for multimodal data. https://www.rerun.io/.

[36] Rohrer B., Fasoli S., Krebs H.I., Hughes R., Volpe B., Frontera W.R. (2002). Movement smoothness changes during stroke recovery. J Neurosci.

[37] Balasubramanian S., Melendez-Calderon A., Roby-Brami A., Burdet E. (2015). On the analysis of movement smoothness. J NeuroEng Rehabil.

[38] Gladstone D.J., Danells C.J., Black S.E. (2002). The Fugl-Meyer assessment of motor recovery after stroke: a critical review of its measurement properties. Neurorehabil Neural Repair.

[39] Hudak P.L., Amadio P.C., Bombardier C., Beaton D., Cole D., Davis A. (1996). Development of an upper extremity outcome measure: the dash (disabilities of the arm, shoulder, and hand). Am J Ind Med.

[40] Murphy M.A., Willén C., Sunnerhagen K.S. (2011). Kinematic variables quantifying upper-extremity performance after stroke during reaching and drinking from a glass. Neurorehabil Neural Repair.

[41] Broeren J., Sunnerhagen K.S., Rydmark M. (2007). A kinematic analysis of a haptic handheld stylus in a virtual environment: a study in healthy subjects. J NeuroEng Rehabil.

[42] Lum P.S., Mulroy S., Amdur R.L., Requejo P., Prilutsky B.I., Dromerick A.W. (2009). Gains in upper extremity function after stroke via recovery or compensation: potential differential effects on amount of real-world limb use. Top Stroke Rehabil.

[43] Teulings H.-L., Contreras-Vidal J.L., Stelmach G.E., Adler C.H. (1997). Parkinsonism reduces coordination of fingers, wrist, and arm in fine motor control. Exp Neurol.

[44] Balasubramanian S., Melendez-Calderon A., Burdet E. (2012). A robust and sensitive metric for quantifying movement smoothness. IEEE Trans Biomed Eng.

[45] Hussain N., Alt Murphy M., Sunnerhagen K.S. (2018). Upper limb kinematics in stroke and healthy controls using target-to-target task in virtual reality. Front Neurol.

[46] Norkin C.C., White D.J. (2016).

[47] Lobbos E., Smith R., Tanaka Y. (2025). Influence of pelvic position on shoulder range of motion. BMC Musculoskelet Disord.

[48] Combettes S.W., Boniol P., Mazarguil A., Wang D., Vaquero-Ramos D., Chauveau M. (2024). Arm-CODA: a data set of upper-limb human movement during routine examination. Image Process Line.

[49] Lento B., Segas E., Leconte V., Doat E., Danion F., Péteri R. (2024). 3D-ARM-gaze: a public dataset of 3D arm reaching movements with gaze information in virtual reality. Sci Data.

[50] Miron A., Sadawi N., Ismail W., Hussain H., Grosan C. (2021). IntelliRehabDS (irds)—a dataset of physical rehabilitation movements. Data.

[51] Karaim M., Noureldin A., Karamat T.B. (2019). 2019 international conference on communications, signal processing, and their applications (ICCSPA).

[52] El-Sheimy N., Nassar S., Noureldin A. (2004). Wavelet de-noising for imu alignment. IEEE Aerosp Electron Syst Mag.

[53] Kj N., Sreejith A.G., Mathew J., Sarpotdar M., Suresh A., Prakash A., Navarro R., Burge J.H. (2016). Advances in optical and mechanical technologies for telescopes and instrumentation II.

[54] Rerun Development Team (2025). Limit the viewer's memory usage. https://rerun.io/docs/howto/visualization/limit-ram.

